# Towards the elimination of *Plasmodium vivax* malaria: Implementing the radical cure

**DOI:** 10.1371/journal.pmed.1003494

**Published:** 2021-04-23

**Authors:** Kamala Thriemer, Benedikt Ley, Lorenz von Seidlein

**Affiliations:** 1 Global and Tropical Health Division, Menzies School of Health Research and Charles Darwin University, Darwin, Australia; 2 Mahidol-Oxford Tropical Medicine Research Unit (MORU), Faculty of Tropical Medicine, Mahidol University, Bangkok, Thailand; 3 Centre for Tropical Medicine and Global Health, Nuffield Department of Medicine, University of Oxford, Oxford, United Kingdom

## Abstract

In this review for the Vivax malaria collection, Kamala Thriemer and colleagues explore efforts to eliminate P. vivax malaria.

**Summary points**

- Efforts to control *Plasmodium vivax* malaria have been less successful than for *Plasmodium falciparum*, resulting in higher prevalence of *P*. *vivax* malaria in most coendemic regions. One of the key differences between the 2 species is the ability of *P*. *vivax* to form hypnozoites causing relapses which facilitate transmission. Preventing *P*. *vivax* relapses is key for the elimination of *P*. *vivax* malaria.
- The widescale use of the radical cure to clear hypnozoites has been underutilized in most endemic countries. Two breakthroughs have increased the likelihood that the radical cure will be rolled out in *P*. *vivax* endemic regions: To clear hypnozoites, primaquine can be administered in short, high-dose regimens or a single dose of the recently licensed tafenoquine is administered. Novel technologies allow measurement of glucose-6-phosphate dehydrogenase (G6PD) activity at the point of care. Identifying patients with low G6PD activity, not eligible for these novel regimens, is a precondition for their safe administration.
- Novel approaches to *P*. *vivax* elimination such as mass drug administrations of antimalarial drugs including 8-aminoquinolines require considerable resources and carry safety risks.
- A safe and protective *P*. *vivax* vaccine would be an asset in the elimination of *P*. *vivax* malaria but is unlikely to be available in the near future.
- Case management that includes a radical cure is currently the most promising approach to *P*. *vivax* elimination. New regimens for radical cure and the possibility to minimise the risk of haemolysis through novel G6PD tests bring up operational challenges, but if deployed wisely could have sufficient impact to eliminate if not eradicate *P*. *vivax* malaria.

## Introduction

**Fig 1:**
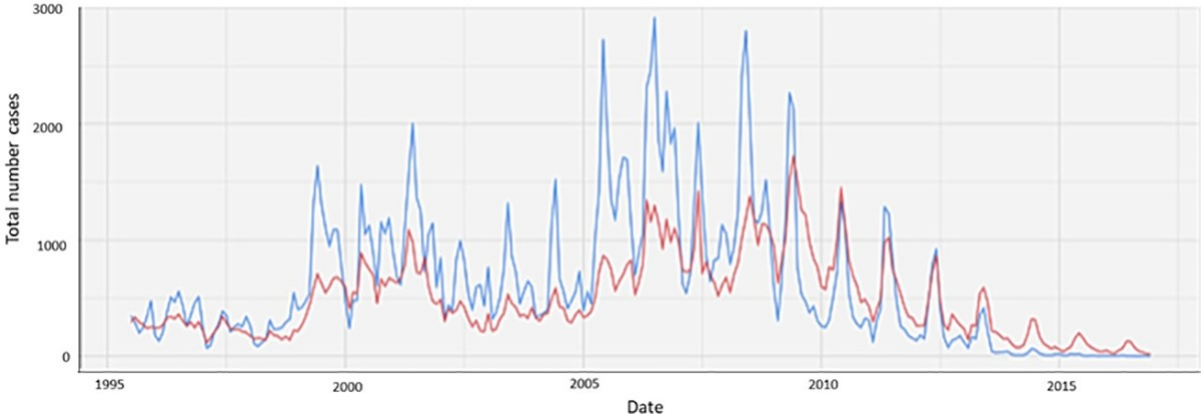
Number of *P*. *falciparum* and *P*. *vivax* cases detected between 1995 to 2016 in the refugee and migrant clinics in the Shoklo Maria Research Unit (SMRU), Thailand. *P*. *falciparum* is indicated in blue and *P*. *vivax* in red. A range of interventions (Figure provided by Cindy Chu [2]).

**Fig 2:**
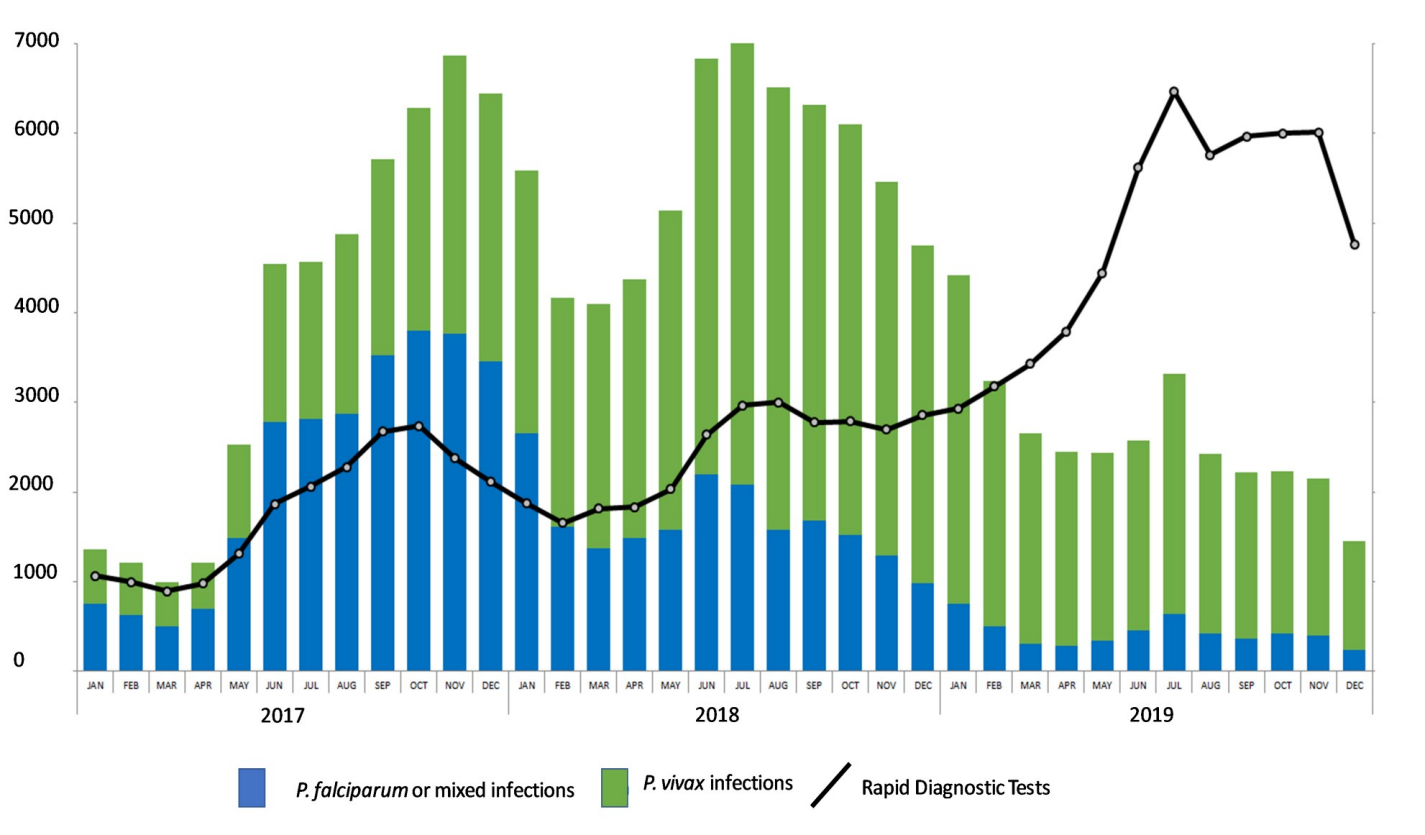
Monthly malaria cases reported by Cambodian National Malaria Control Programme CNMCP information system between 2017–2019 [3]. The number of rapid diagnostic tests (RDTs) increased while the number of diagnosed cases dropped.

The impact of malaria control interventions has been more pronounced on *Plasmodium falciparum* than *Plasmodium vivax.* In many places where *P*. *falciparum* and *P*. *vivax* malaria coexist, the *P*. *vivax* burden is now larger than the burden caused by *P*. *falciparum* [1]. Following the implementation of antimalarial measures along the Thai-Myanmar border, all malarias decreased, but the effect was greater on *P*. *falciparum* than *P*. *vivax* malaria (Fig 1) [2]. Similarly, successful control of falciparum malaria concomitant with delays in the reduction of *P*. *vivax* has been reported in Cambodia (Fig 2), Papua New Guinea, Costa Rica, and Brazil [3–6]. Globally, the number of *P*. *vivax* malaria cases nevertheless decreased from 25 million in 2000 to 14 million in 2017, and the number of countries reporting locally acquired cases of *P*. *vivax* decreased from 58 in 2000 to 49 in 2013 [1,7].

*P*. *vivax* is more difficult to control than *P*. *falciparum* due to its ability to form dormant liver forms (hypnozoites). Even after clearing *P*. *vivax* schizonts from the bloodstream, infected individuals may experience relapses due to activation of hypnozoites and become sources of transmission. In tropical regions, 4 of 5 *P*. *vivax* patients have early relapses occurring every 3 to 4 weeks. With increasing distance from the equator, the relapse risk decreases and the latency period increases, which can be as long as 8 to 13 months closer to the arctic circle [8–10]. Control and elimination of *P*. *vivax* requires treatment of all life-stages of *P*. *vivax* (radical cure). Hypnozoites can only be cleared by 8-aminoquinolines, a class of drugs which can trigger haemolysis in glucose-6-phosphate dehydrogenase (G6PD)-deficient individuals [11].

Across the Asia-Pacific region and the Horn of Africa between 66% and 95% of acute episodes are estimated to be caused by relapse [12,13]. The radical cure can prevent relapse and is therefore expected to play a pivotal part in the elimination efforts [14]. This paper discusses current evidence and challenges for the radical cure in the context of malaria elimination and progress of alternative strategies.

### Radical cure: Current policy and new approaches

Current World Health Organization (WHO) guidelines recommend coadministration of schizonticidal treatment with a low-dose primaquine (PQ) regimen (total dose 3.5 mg/kg at 0.25 mg/kg/day) for temperate strains and a high-dose PQ course (total dose 7 mg/kg at 0.5 mg/kg/day) for tropical, frequent-relapsing strains administered over 14 days. Routine testing of G6PD deficiency (G6PDd) prior to drug administration is recommended by global policies; however, it is rarely available in poorly resourced communities [11]. To mitigate the risk of haemolysis, many countries in which the higher dose is recommended, opt for the low-dose course of PQ (Table 1).

**Table 1:** Overview of current policy recommendation by country based on WHO 2018 World Malaria report.

	Current treatment recommendation for *P*. *vivax*		Recommendation for Directly Observed Treatment Strategy		G6PD testing	
Country (WHO region)	Drug and PQ dosage	Year of policy adoption	DOTs for PQ recommended	Year of policy adoption	G6PD testing recommended	Year of policy adoption
Africa						
Ethiopia	CQ+PQ^1^	2018	No		No	
Eritrea	AS+AQ+PQ (14 d 0.25 mg/kg)	2007	Yes	2016	No	
Madagascar	PQ (14 d 0.25 mg/kg)^2^	-	Yes	2015	No	
South Sudan	AS+AQ+PQ^1^	-	No		No	
Americas						
Brazil	CQ+PQ (7 d 0.5 mg/kg)	2006	No		No	
Belize	CQ+PQ (14 d 0.25 mg/kg)	-	Yes	-	No	
Bolivia	CQ+PQ (14 d 0.25 mg/kg)	2001	No		No	
Colombia	CQ+PQ (14 d 0.25 mg/kg)	1960	Yes	2012	No	
Costa Rica	CQ+PQ (14 d 0.5 mg/kg)	2008	Yes	1957	No	
Ecuador	CQ+PQ (7 d 0.5 mg/kg)	2004	No		No	
French Guyana	CQ+PQ (14 d 0.5 mg/kg)	-	No		Yes	-
Guatemala	CQ+PQ (14 d 0.25 mg/kg)	-	No		No	
Guyana	CQ+PQ (14 d 0.25 mg/kg)	2004	No		No	
Honduras	CQ+PQ (14 d 0.25 mg/kg)	2011	No		No	
Nicaragua	CQ+PQ (7 d 0.5 mg/kg)	-	Yes	1980	No	
Panama	CQ+PQ (14 d 0.25 mg/kg)	-	Yes	-	No	
Peru	CQ+PQ (7 d 0.5 mg/kg)	2001	Yes	1994	No	
Suriname	CQ+PQ (14 d 0.25 mg/kg)	2004	No		No	
Venezuela	CQ+PQ (14 d 0.25 mg/kg)	2004	No		No	
Eastern Mediterranean						
Afghanistan	CQ+PQ (8 w 0.75/kg)	2014	No		Yes	2017
Djibouti	AL+PQ (14 d 0.25 mg/kg)	-	No		No	
Iran	CQ+PQ (8 w 0.75 mg/kg)	-	Yes	1949	No	
Pakistan	CQ+PQ (14 d 0.25 mg/kg)	2017	No		Yes	2016
Somalia	AL+PQ (14 d 0.25 mg/kg)	2014	Yes	2016	Yes	2016
Sudan	AL+PQ (14 d 0.25 mg/kg)	-	No		No	
Yemen	CQ+PQ (14 d 0.25 mg/kg)	-	No		Yes	2009
Southeast Asia						
Bangladesh	CQ+PQ (14 d 0.25 mg/kg)	2004	No		No	
Bhutan	CQ+PQ (14 d 0.25 mg/kg)	2006	No		No	
North Korea	CQ+PQ (14 d 0.25 mg/kg)	-	Yes	2000	No	
India	CQ+PQ (14 d 0.25 mg/kg)	2007	No		No	
Indonesia	DHA-PQ+PQ (14 d 0.25 mg/kg)	2008	No		No	
Myanmar	CQ+PQ (14 d 0.25 mg/kg)	2002	Yes	2017	Not yet	2021
Nepal	CQ+PQ (14 d 0.25 mg/kg)	2004	No		Yes	-
Thailand	CQ+PQ (14 d)^1^	2007	Yes	2011	Yes	2015
Timor-Leste	CQ+PQ (8 w 0.75 mg/kg)	-	Yes	2016	Yes	2016
Western Pacific						
Cambodia	AS+MQ+PQ (14 d 0.25 mg/kg)	2011	No		Yes	2012
China	CQ+PQ; ACTs+PQ (8 d 0.75 mg/kg)	2016	Yes	1970	No	
Lao People’s Democratic Republic	AL+PQ (14 d 0.25 mg/kg)	2017	No		Yes	2010
Malaysia	CQ+PQ (14 d 0.5 mg/kg)	2016	Yes	1993	Yes	1993
Papua New Guinea	AL+PQ (14 d 0.25 mg/kg)	2009	No		No	
Philippines	CQ+PQ (14 d 0.25 mg/kg)	2002	Yes	2010	Yes	2011
Republic of Korea	CQ+PQ (14 d 0.25 mg/kg)	1997	No		No	
Solomon Islands	AL+PQ (14 d 0.25 mg/kg)	2009	No		Yes	2009
Vanuatu	AL+PQ (14 d 0.25 mg/kg)	2007	Yes	2009	Yes	2009
Vietnam	CQ+PQ (14 d 0.25 mg/kg)	2016	Yes	2014	No	

^1^No PQ dosage provided.

^2^No information on schizontocidal treatment provided.

ACT, artemisinin combination therapy; AL, artemether–lumefantrine; AQ, amodiaquine; AS, artesunate; CQ, chloroquine; DHA-PQ, dihydroartemisinin–piperaquine; DOTS, directly observed treatment strategy; G6PD, glucose-6-phosphate dehydrogenase; PQ, primaquine.

When supervised, this low-dose regimen has an efficacy greater than 70% at 6 months in some, but not all, locations [15,16]. However, it can be challenging to ensure adherence to a 14-day treatment regimen, especially since initial symptoms in most cases resolve within 2 days after start of effective schizontocidal treatment. Adherence is critical for radical cure, since efficacy is related to the total dose of PQ administered [15,17]. While efficacy is important, effectiveness which takes into account adherence and the limitations outside of carefully controlled trials is the key measure needed to inform malaria elimination strategies. A prospective analysis in 48,000 patients with *P*. *vivax* presenting to a district hospital in Papua Indonesia, demonstrated that an unsupervised 14-day PQ regimen prevented recurrent parasitaemia within 1 year in only 10% of the participants [18]. This low effectiveness is likely due to poor adherence to the 14-day treatment course [19,20]. Direct comparisons of supervised versus unsupervised treatment support this notion [21–23]. In a recent study in Ethiopia, patients treated with unsupervised 14-day PQ were at significantly greater risk of recurrent *P*. *vivax* parasitaemia within 6 months of follow-up than those in which treatment was semi-supervised [21]. Similar findings were reported in Costa Rica where PQ treatment is supervised by trained personnel [14,24]. Elimination is possible with optimal use of current tools as demonstrated by Sri Lanka which has recently been declared malaria free [25].

### Higher PQ doses and shorter treatment regimens

Although a high-dose PQ regimen is recommended for some areas, there is limited evidence in which locations and host populations it might be most beneficial and where a low-dose regimen might provide adequate cure [26,27]. To date, only 5 published studies have compared low- versus high-dose PQ directly, and findings are limited due to small numbers and short follow-up periods [28–32]; further work is ongoing [33]. A recent multicentre study investigated whether a high-dose PQ regimen administered over 7 days (1 mg/kg/day) was non-inferior to the same total dose given as a 14-day regimen (0.5 mg/kg/day). A total of 2,388 patients were enrolled and followed for 12 months in sites in Ethiopia, Indonesia, Vietnam, and Afghanistan [34,35]. There was no significant difference in the risk of *P*. *vivax* recurrence between the 7- and the 14-day high-dose regimens, and at 1 year, the overall risk of recurrence was 13% following high-dose PQ. At the Ethiopian study site, the risk of recurrence at 6 months was almost half compared to a low-dose regimen conducted 2 years previously (10% versus 17%) [21,36]. With higher daily doses, the concerns over potential drug-induced haemolytic events increase. In this study, 935 of the 2,388 patients enrolled were treated with high-dose PQ. Four (4/935) haemolytic events occurred, one in a patient who was erroneously enrolled into the study despite being G6PD deficient and required a blood transfusion. The other 3 patients recovered rapidly after stopping the trial medication [36]. Complementary data from a Thai study demonstrated that heterozygous females with intermediate G6PD activity were at greater risk from haemolysis following a treatment with 1 mg/kg PQ per day than patients who received 0.5 mg/kg/day; however, this was only clinically relevant in 2 patients, both of whom manifested symptoms at day seven [37]. Studies assessing even shorter high-dose PQ courses with 1.0 mg/kg twice daily dose (bd) for a total of 3.5 days are ongoing and if found to be safe and effective could shorten treatment regimens even further [38].

### Tafenoquine

Tafenoquine (TQ), like PQ, is an 8-aminoquinoline drug but is eliminated much slower than PQ (14 to 28 days versus 4 to 6 hours). TQ administered as a single dose provides a significant advantage for adherence but, in contrast to PQ treatment, cannot be curtailed when signs and symptoms of haemolysis are detected. A recent trial using 300 mg single-dose TQ found that 31% of patients had *P*. *vivax* recurrence at 6 months compared to 24% in a low-dose PQ arm [16]. Major differences in efficacy between the study sites suggests that a 300-mg TQ regimen is suboptimal in regions where higher PQ concentrations are required to clear hypnozoites [39]. Declines in haemoglobin following TQ administration of more than 3*g* per decilitre or at least 30% of baseline levels were uncommon and did not require clinical intervention [40]. Initially TQ has been licensed for use with any schizontocidal treatment. A recent label change to administer TQ exclusively with chloroquine and no other schizonticidal drug has limited its usefulness. TQ has been licensed for use in G6PD normal patients, defined as a G6PD enzyme activity greater than 70%, and its use is currently still restricted to patients above 16 years of age. These restrictions, unique to TQ, make it unlikely that TQ will replace PQ at least in the short term [41].

### Radical cure in G6PD-deficient individuals

Current WHO guidelines for patients with mild to moderate G6PDd recommend weekly PQ doses of 0.75 mg/kg over 8 weeks following a careful risk–benefit assessment taking into consideration the availability of close medical supervision and access to transfusion services [42]. There is limited data on the safety of this regimen and more recent data from Cambodia suggest caution as G6PDd patients experienced significant, although transient, drops in haemoglobin levels following the weekly PQ administration [43]. More data are required to make evidence-based treatment decisions for this neglected patient population.

### Universal radical cure

There is a growing body of evidence for an increased risk of *P*. *vivax* parasitaemia following falciparum malaria, higher than would be expected from the risk of reinfection alone [29,44–46]. In coendemic areas, patients presenting with *P*. *falciparum* may have had prior infections with *P*. *vivax*, present either at undetectable levels in the peripheral blood and/or dormant hypnozoites. Both fever and haemolysis associated with malaria have been hypothesised to stimulate the reactivation of hypnozoites resulting in relapsing infections [8,47,48]. In a retrospective pooled analysis of 10,549 patients with uncomplicated *P*. *falciparum* malaria treated on the Thai-Myanmar border, the cumulative proportion of patients with *P*. *vivax* recurrence was 31.5% by day 63 [44]. A more recent systematic analysis including 153 *P*. *falciparum* efficacy studies and a total of 31,262 patients showed that the risk of *P*. *vivax* parasitaemia was greater in regions of short relapse periodicity and after more rapidly eliminated artemisinin-based combination therapy (ACT) reaching 15.3% after *P*. *falciparum* treatment with artemether-lumefantrine [45]. The risk of recurrent *P*. *vivax* malaria episodes provides a strong rationale for universal use of radically curative treatment in patients with *P*. *falciparum* malaria even in the absence of detectable *P*. *vivax* parasitaemia in areas that are coendemic for these species. A review of patient data from the Thai-Myanmar border over 7 years showed a decreasing risk of *P*. *vivax* recurrence after initial falciparum infection from more than 20% in 2003 to below 5% in 2010, suggesting that overall improvements in malaria control led to a reduction of coinfected patients [46]. Prospective studies to evaluate the potential benefit of universal radical cure in different locations are currently ongoing (NCT 03916003) and could represent a cost-effective approach to clear otherwise unrecognised *P*. *vivax* infections and hence accelerate *P*. *vivax* elimination.

### The quest for the right G6PD test

The single most important constraint on the global deployment of 8-aminoquinolines is their potential to cause haemolysis in patients with low G6PD activity. Low G6PD activity, commonly known as G6PDd, is among the most prevalent enzymopathies worldwide [49]. In practice, the lack of available robust diagnostics for G6PDd, fear of drug-induced haemolysis, the perceived benign nature of *P*. *vivax* infection, and fear of additional costs to health systems have hindered a broad roll out of routine G6PD testing to guide *P*. *vivax* radical cure [50].

The *G6PD* gene is located on the X-chromosome (Xq28); males harbor 1 copy of the gene and are either G6PD deficient or normal, females harbor 2 *G6PD* copies and can be homozygous deficient or normal or heterozygous for the gene [51]. Heterozygous females are discriminated at a 60% to 80% cut-off activity; this can only be determined by quantitative diagnostics. In contrast, hemizygous males and homozygous females have G6PD activities below 30% activity, the optimal cut-off for most, if not all, qualitative diagnostics [52–54].

G6PD activity is measured in U/gHb, but no universal cut-off for G6PDd has been defined, not least because standardization of the phenotypic reference method, spectrophotometry, is poor [55]. Instead, 100% activity is defined based on the adjusted male median (AMM) G6PD activity [56]. This definition is site, assay, and population specific and needs to be established prior to routine use of quantitative diagnostics. In contrast, the variation around the 30% threshold is smaller, hence qualitative assays can be implemented without prior assessment of the target population. Depending on radical cure regimen, cut-off activities that guide treatment decision vary between 30% to 70%, the choice of the best G6PD test format varies accordingly, depending on treatment regimen and sex of the patient [16,42,56,57].

Quantitative UV-spectrophotometry, the phenotypic reference method, is costly and requires a well-established laboratory infrastructure (Table 2) [55,56,58]. The spectrophotometry output requires calculation using the corresponding haemoglobin reading to derive a result in U/gHb, the necessary format to guide treatment. More recently, a number of handheld biosensors have been developed with superior operational characteristics to spectrophotometry; however, only 1 device (G6PD Standard, SDBiosensor, Korea) fits currently established target product profiles [59–62]. Performance of the Biosensor in routine care is yet to be assessed (Table 2).

**Table 2:** Glucose-6-phosphate dehydrogenase deficiency tests suitable for field use.

Assay	Type	Manufacturer	Sensitivity (30% cutoff, 70% cutoff)	Specificity (30% cutoff, 70% cutoff)	Price* (USD)	Additional requirements	Pipetting steps**	Reference
Spectrophotometry	Quantitative	Multiple	Variable (Reference method)	Variable (Reference Method)	10.00	Spectrophotometer, Hb measurement, Fridge, Water-bath	3	[55,56]
G6PD Standard	Quantitative	SDBiosensor (Korea)	100.0, 90.0–97.2	97.0–100.0, 87.0–97.0	350 / machine, 3.5 / strip	none	2	[59,60]
Fluorescent spot test	Qualitative	Multiple	97.9–100.0, 71.9–80.0***	71.1–90.1, 82.0–91.1	5.50–14.00	Fridge, Water-bath, UV-Lamp	2	[64,95]
Carestart G6PD screening test	Qualitative	Accessbio-Carestart (USA)	96.0, ND	95.0, ND	1.50	none	1	[56,65]

ND, No data.

*Prices are approximate, vary significantly from country to country, and exclude required hardware and labor costs.

**As proxy for complexity, not considering preparation of supplies.

***Considering intermediate results as deficient.

The fluorescent spot test (FST) is probably the most widely used qualitative G6PD diagnostic test since its introduction in the late 1960s [63]. When performed under optimal conditions, the FST has a sensitivity of above 95% at a 30% cut-off, but the test requires refrigeration of supplies, a water bath, a UV-lamp, and an experienced tester, rendering the test unsuitable for primary care settings [64]. A number of qualitative assays have been introduced to the market over the last couple of years; however, only a lateral flow assay from Accessbio/Carestart (United States of America) shows operational and performance characteristics suitable to guide radical cure in routine practice [62,65,66] (Table 2). No molecular assays for point-of-care G6PD testing are currently on the market; respective assays would need to be tailored to specific populations and would ideally diagnose multiple variants simultaneously [67].

In *P*. *vivax* malaria endemic settings with limited resources for G6PD testing, recording the result of a single G6PD diagnosis for future treatment is appealing. However, G6PD activity is subject to variation, the extent of which is unknown. G6PD activity varies with age of the red blood cell (RBC) population, with reticulocytes and younger RBC having higher G6PD activity [68–71]. Malaria induces haemolysis, resulting in reactive erythropoiesis, a decrease in the age of the RBC population and consequently an increase of G6PD activity [71–74]. In a multicentre trial in 6 African countries, a total of 42 out of 124 (19.4%) participants hemi- or homozygous for the A- G6PD variant were phenotypically intermediate or normal when diagnosed by FST [75]. A transitional increase in G6PD activity is likely to protect against drug-induced, severe haemolysis, but this assumption needs to be confirmed in real-life settings [71]. Some countries have introduced neonatal screening for G6PD deficiency to identify newborns at high risk for kernicterus. Such data could theoretically guide treatment decisions for *P*. *vivax* malaria later in life [76]. However, studies have shown that neonatal G6PD activity is significantly higher compared to adult activities [77]. These findings suggest that G6PD testing is ideally done directly prior to each radical cure treatment.

### How to roll out the radical cure?

There are 4 main issues that need to be considered in each setting to determine optimal radical cure strategies: (i) which drug regimen is the most suitable; (ii) which G6PD test is required for this treatment; (iii) what other measures are available to ensure patient safety; and (iv) where and by whom (e.g., at which level of the healthcare system) will radical cure be provided.

More than half of all patients with *P*. *vivax* malaria live in remote and rural areas. This adds challenges, given that the majority of patients with *P*. *vivax* malaria only have access to primary healthcare, and most resource-poor settings have limited functioning mechanisms to transfer patients to higher-level facilities. The safe delivery of a radical cure is not only a function of adequate testing, as no test will have 100% sensitivity and specificity and mistakes are likely to happen in routine setting. Even in well-resourced and tightly controlled clinical trials, a G6PD-negative patient mistakenly received high-dose PQ [36,78]. Safe radical cure depends on a functioning healthcare system including adequate capacity for pharmacovigilance. Measures to increase patient safety, including adequate follow-up that increases the chance of detecting potential adverse events early and mitigating them appropriately (e.g., stopping PQ treatment), are gaining importance.

Where and by whom the radical cure should be provided is ultimately an operational question for control programmes and answers will largely depend on the drug regimen, where the chosen G6PD test can be performed and interpreted and where the majority of cases are located. Achieving large impact will most likely require administering the radical cure at the community level and not only in higher-level healthcare centres to ensure immediate access for all affected people. The exception are countries with extremely low numbers of cases and with well-functioning patient transfer mechanisms to higher-level healthcare or those aiming to avoid reintroduction. The majority of countries will need to make use of community health workers for the effective roll out of the radical cure. Community-delivered healthcare has shown to be effective in reducing overall malaria-associated mortality yet their differential impact on malaria-metric outcomes is difficult to assess [79] and most work on community-delivered models has focused on falciparum malaria [79,80]. Major barriers to the roll out of radical cure at community level are concerns over the capacity and training needs for correct G6PDd testing, adherence to test and treatment algorithms, as well as the capacity to detect adverse events, stop further doses of PQ and provide blood transfusions, when needed. Operational research in this area has largely focused on G6PD testing, including user-friendliness, acceptability, feasibly, training needs, and procurement issues, but less on broader health system issues or actual delivery mechanism [81–83]. It remains to be explored what delivery model is best suited to provide safe radical cure and what level of training and supervision might be required. Where radical cure is delivered at the community level, patient transfer systems to higher-level care facilities have to be available. Alternatively, radical cure could be provided by mobile clinics and community-based healthcare workers provide longer-term follow-up. Future WHO treatment guidelines are likely to include broad recommendations on the use of novel tools for *P*. *vivax* malaria; however, they will require adaptation for each national and or subnational level taking the discussed issues into consideration.

### Alternative approaches to *P*. *vivax* elimination: Vaccines and mass drug administrations

The falciparum vaccine development has been slow, and the development of vaccines to protect against *P*. *vivax* is lagging further behind. A vaccine targeting the *P*. *vivax* circumsporozoite protein PvCSP, the orthologue of the PfCSP targeted by RTS,S/AS01, also adjuvanted by AS01, did not protect against a challenge with *P*. *vivax* sporozoites [84]. *P*. *vivax* binds to the Duffy antigen receptor for chemokines (DARC) via a Duffy binding protein (PvDBP) which has been the target for 2 blood stage vaccines which are both in early development [84,85]. There is promising ongoing research for *P*. *vivax* vaccine targets and vaccination strategies, but the chances for the rollout of a *P*. *vivax* vaccine in the foreseeable future are minimal.

In the absence of a long-lasting, protective vaccine, researchers have experimented with mass administrations of antimalarial drugs to eliminated malaria. To make an impact on transmission, such mass drug administrations (MDAs) have to cover the entire or nearly the entire population in the target area because malaria has a high basic reproductive number [86]. To engage the community in such an undertaking is time and resource intensive. Past MDAs in regions surrounded by ongoing *P*. *falciparum* malaria transmission have succeeded to suppress transmission for limited periods but failed to interrupt transmission permanently probably due to residual parasite reservoirs and reimportation of infections [87]. In the presence of residual hypnozoites, the suppression of *P*. *vivax* prevalence is even shorter than *P*. *falciparum* prevalence [88]. To make a lasting impact, MDAs aiming to eliminate *P*. *vivax* malaria have to include 8-aminoquinolines. During the Soviet era, 8,270,185 people in Azerbaijan, Tajikistan, North Afghanistan, and DPR Korea received either a 14-day “standard” or a 17-day “interrupted” PQ treatment to control post-eradication malaria epidemics [89]. The reported frequency of severe adverse events related to PQ was considered “very low.” Between 1973 and 2009, people living in Jiangsu Province, China received 154,826,505 courses of MDA which included PQ (22.5 mg) for 8 days [25]. In 2 counties where adverse events were recorded, the incidence of acute haemolysis was 3.5 and 9.3 per 100,000 population, respectively. Today, few policymakers are willing to accept such a risk even if these are small. More recently, MDA of antimalarial drugs including PQ have been conducted in populations thought to be free of G6PDd, e.g., Nicaragua and Costa Rica [6,90]. In Aneityum, the southernmost island of Vanuatu, a 9-week regimen of weekly PQ doses thought to be safe in people with G6PDd succeeded in interrupting malaria transmission for extended periods [91]. Yet few populations at risk for *P*. *vivax* malaria are thought to be free of G6PDd, and 9 weekly doses of PQ limit the generalizability of this approach. Using TQ in MDAs would be attractive as a single dose regimen is likely to result in a higher coverage than multidose regimens. Mathematical modelers, free of operational constraints, predict that TQ with screening for G6PDd administered as part of first-line treatment or through MDA could result in a 58% to 86% reduction in *P*. *vivax* cases in Papua New Guinea [92]. A novel addition to the antimalarial regimens used in MDA is ivermectin. Field studies have shown an added killing effect of mass ivermectin administrations against malaria vectors and could contribute to a long-lasting impact against all malarias [3,93,94].

## Conclusions

There is a broad consensus in the malaria community that early diagnosis and treatment is key to malaria elimination. This strategy has been less successful for *P*. *vivax* control than for *P*. *falciparum* as evidenced by more recent relative increases in the *P*. *vivax / P*. *falciparum* ratios in many coendemic countries. The most important reason for this difference is *P*. *vivax*’s propensity to relapse and the limited therapeutic options to tackle the hypnozoite reservoir. Two recent developments provide hope that the radical cure and, hence, the clearance of hypnozoites, will become available more broadly. First, TQ, a long-acting 8-aminoquinoline, has been licensed, and efforts for roll out in endemic countries are underway. Similarly, shorter courses of PQ are expected to increase effectiveness and could provide alternatives to current treatment options. Second, rapid point-of-care tests have become available which may allow G6PD testing in remote settings, reducing the need to transfer patients to higher-level healthcare facilities for radical cure.

These developments have brought up 4 major questions for the elimination of *P*. *vivax* malaria: What is the most appropriate 8-aminoquinoline regimen in a given setting, what is the most appropriate G6PD test, what other measures are available to ensure safety, and where and by whom should the radical cure be administered? Finding the optimal solutions for the operational challenges implied in each of these questions is likely to hold the key for *P*. *vivax* elimination. The implementation of those solutions will require national, regional, and global leadership and the continued commitment to sustained funding from national and global funding bodies and national governments.

## Abbreviations

ACT: artemisinin-based combination therapy
AMM: adjusted male median
DARC: Duffy antigen receptor for chemokines
FST: fluorescent spot test
G6PD: glucose-6-phosphate dehydrogenase
G6PDd: G6PD deficiency
MDA: mass drug administration
PQ: primaquine
RBC: red blood cell
TQ: tafenoquine
WHO: World Health Organization
